# Evaluation of replication success: A statistical perspective and extended analysis of the SCORE data

**DOI:** 10.1371/journal.pone.0358126

**Published:** 2026-09-30

**Authors:** Robbie C. M. van Aert, Marcel A.L.M. van Assen, Rubén López-Nicolás

**Affiliations:** 1 Department of Methodology and Statistics, Tilburg University, Tilburg, Netherlands; 2 Universidad de Murcia, Murcia, Spain; University of Sao Paulo: Universidade de Sao Paulo, BRAZIL

## Abstract

Replications are more and more conducted in multiple research fields. A recent large-scale replication project, Systematizing Confidence in Open Research and Evidence (SCORE) project, replicated 274 effects from research on business, economics, education, political science, psychology, and sociology. An open question remains how replication success can best be assessed. Thirteen methods for assessing replication success were applied in the SCORE project. The first goal of this paper is studying the statistical properties of replication success methods by a numerical study tailored to the characteristics of the SCORE data. The results of the numerical study show that replication success methods are unable to reliably draw a conclusion about replication success for typical sample sizes of the original study and replication. Hence, there is often insufficient information in one original study and one replication for drawing definite conclusions. The second goal of this paper is taking a novel perspective on the SCORE data by estimating the effect size of an original study and replication. Estimators based on meta-analysis methods that correct for potential bias in the original study were close to unbiased when synthesizing the original study and replication. The reanalysis of the SCORE data revealed that meta-analytic estimates were substantially closer to the effect size of the replication compared to the original study. The null hypothesis of no effect was rejected by the meta-analysis methods in 59.2% to 81.3% of the pairs of an original study and replication. We recommend researchers to make sure that methods for analyzing replicability have favorable statistical properties for the data at hand before applying these methods.

## Introduction

More and more replications are conducted in psychological research [1]. The main goal of a replication is usually to examine the replicability of a study, here defined as whether the same result is obtained as in the original study if the original study is executed again [2]. A single replication of an original study is sometimes conducted as well as large-scale replicability projects where the focus is on studying the replicability of multiple original studies of one or more research fields [3–6]. Other replicability projects focused on replicating a single original study in multiple labs to study the generalizability of multiple effects [i.e., Many Labs projects, [7–10].

The overall conclusion of the replicability projects in psychology, economics, and cancer biology is that the replicability of studies is low [3–6]. However, the assessment of the replicability of studies (i.e., is the original study successfully replicated?) is difficult. Currently, there is no consensus on how to best assess replicability. Common approaches used when studying replicability are whether the effect size of the original study and replication are in the same direction, the original study and replication are both statistically significant and in the same direction, or the effect size of the original study is in the 95% confidence interval (CI) of the replication. There are more approaches to study replicability based on, for example, meta-analysis methods [11–15], the prediction interval (PI; [16]), statistical power analysis [17], combining null hypothesis significance testing and equivalence testing [18], Bayes factors [19], and reverse Bayes analysis [20].

The statistical properties of a selection of the approaches to assess replicability have already been studied in a series of papers by Schauer, Hedges, and colleagues [12,21–26] and in Muradchanian et al. [27], Freuli et al. [28], and Kondzic et al. [29]. The overarching conclusion based on the studies by Schauer, Hedges, and colleagues is that the common approaches used in these replicability projects have unfavorable statistical properties such as a high Type-1 error rate. They also conclude that one original study and one replication is often not enough to have high statistical power when testing for replicability. In a simulation study, Muradchanian et al. [27] examined the statistical properties of multiple approaches to study replicability when the original study is affected by publication bias. Their results show that Bayesian methods and meta-analysis methods had the best properties when looking at the true and false positives. Freuli et al. [28] extended this research by also including different types of questionable research practices in the original study. Especially the questionable research practices selectively reporting outcomes and subgroup analyses yielded larger false positive rates of all included methods to assess replicability. Kondzic et al. [29] also conducted a simulation study but focused, in contrast to Muradchanian et al. [27] and Freuli et al. [28], on a prospective replication design. In a prospective replication design, the original study and replication are planned simultaneously and the design of the replication does not depend on the results of the original study. Kondzic et al. [29] concluded that there is also no single best replication success method for prospective replication designs and that Type-1 error rates can be inflated.

The difficulties of assessing replicability based on only one original study and one replication have also motivated to focus on answering the two questions “what is the best estimate when meta-analyzing the original study and replication” and “is the meta-analytic effect different from zero?” These two questions have also been answered in the large-scale replicability projects by fitting a fixed-effect (FE) meta-analysis model [30]. In this FE model (also known as common-effect or equal-effect model), it is assumed that the true effect size of the original study and replication is the same. Answering these two questions about the meta-analytic effect size is arguably also more of substantial interest for the applied researcher. Van Aert et al. [13,14] developed methods for answering these two questions that take into account that the original study is selected; for instance, because replications usually only set out to replicate statistically significant original studies and/or because of publication bias [31]. Their results show that not taking this selection bias into account yield overestimated estimates in the FE model and inaccurate statistical inference while the proposed methods outperform the FE model. However, the results also show, in line with those of Schauer, Hedges, and colleagues, that only one original study and one replication is a challenging condition for the proposed methods that leads to, for example, low statistical power for testing the null hypothesis of no effect and imprecise estimates.

The focus in this paper is on the replicability component of the Systematizing Confidence in Open Research and Evidence (SCORE) project [32]. In this project initiated by the Center for Open Science, the replicability [32], reproducibility [33], and analytical robustness [34] of the literature in business, economics, education, political science, psychology, and sociology were studied. Papers under scrutiny were selected from a sample of 62 journals in these six research fields and within the timeframe of 2009–2018 (see [35] for more details about the sampling). Replicability was studied by matching the selected papers with research teams that had sufficient expertise to replicate it. 274 effects from 164 papers were replicated by either collecting new data or using already existing data that was not incorporated in the original study.

This paper makes both a methodological and empirical contribution to the literature. The methodological contribution is that we assess the performance (i.e., statistical properties) of methods to assess replication success of studies. The assessment of these replicability methods is tailored to data characteristics of the SCORE data such that it is directly applicable to these data. It is currently unknown whether the characteristics of the SCORE data (i.e., one replication study and with sample sizes that are common in these data) allow for drawing conclusions about replication success.

The empirical contribution of our paper is that we reanalyze the SCORE data by taking a different perspective than the analyses in Tyner et al. [32]. They examined the replication success of the studies in SCORE, whereas we also take the perspective by focusing on estimating the meta-analytic effect size when combining the original studies and replications. This will yield relevant insights about, for example, the differences between meta-analytic effect sizes when there is and is not corrected for potential bias in the original studies. Moreover, our analysis also allows for drawing conclusions about differences in meta-analytic effect sizes between research fields. We also first study the statistical properties of these methods before applying the methods to the data of SCORE.

Our paper differs from previous papers on methods to assess replicability and meta-analyzing original studies and replications in three ways. First, the main focus of our paper is to study the statistical properties of methods tailored to the characteristics of the data in SCORE. Previous papers studied the statistical properties by, for example, simulating data according to a particular research field and then evaluate the statistical properties of replication success and meta-analysis methods. However, these simulated data, and consequently also the conclusions of these papers, may not be readily applicable to the data of SCORE. We systematically examine the effect of selection bias, as observed in the SCORE data, on methods for assessing replication success and effect size estimation. The second difference between our paper and previous research is that we also evaluate the statistical properties of methods that aim to estimate the true effect size underlying both the original study and replication, and also in conditions of the SCORE data. Finally, we evaluate the statistical properties of a larger number of methods than what has been done before. These methods were selected because they were applied to the SCORE data [32] or allow estimation and testing of the meta-analytic effect size (see Tables 1 and 2 for an overview).

**Table 1:** Overview of the included methods to assess successful replication.

Method	Description	When is replication success concluded?
**Methods assessing consistency between the conclusions of the original study and replication**		
Agreement in sign and statistical significance	The method is based on the direction of ${\hat{\theta }}_{O}$ and ${\hat{\theta }}_{R}$ and testing ${\text{H}}_{0}:\theta_{O}$ = 0 and ${\text{H}}_{0}:\theta_{R}=0$	If ${\text{H}}_{0}:\theta_{O}$ = 0 and ${\text{H}}_{0}:\theta_{R}=0$ are both rejected (using α=0.05) in combination with ${\hat{\theta }}_{O}$ and ${\hat{\theta }}_{R}$ having the same sign
Sceptical *p*-value	A sceptical prior is defined using ${\hat{\theta }}_{O}$ and its standard error. This sceptical prior is then contrasted with the replication data to evaluate whether the two are in conflict.	If the null hypothesis of no prior-data conflict is rejected with a two-tailed test using the default ’golden’ sceptical *p*-value. This implies that the null hypothesis is rejected for a sceptical *p*-value less than 0.123.
Replication Bayes factor	Comparison of models $\theta_{R}=0$ and $\theta_{R}\sim$ ’posterior distribution original study’	If the Bayes factor of comparing the model $\theta_{R}\sim$ ’posterior distribution original study’ with the model $\theta_{R}=0$ is larger than 3
**Methods assessing similarity of $\theta_{O}$ and $\theta_{R}$**		
Confidence interval (CI)	A 95% confidence interval for $\theta_{R}$ is computed, and it is examined whether ${\hat{\theta }}_{O}$ is in the interval	If ${\hat{\theta }}_{O}$ is in the 95% confidence interval for $\theta_{R}$
Prediction interval (PI)	A 95% prediction interval for $\theta_{O}$ is computed using the square root of the sum of the original and replication variances as the standard error. It is then assessed whether ${\hat{\theta }}_{R}$ falls within this interval.	If ${\hat{\theta }}_{R}$ is in the 95% prediction interval for $\theta_{O}$
Hedges’ *Q*	The *Q*-test used in meta-analysis for testing the null hypothesis of homogeneous true effect size	If the *Q*-test for testing exact replication is not statistically significant using α=0.05
Equivalence test	The equivalence test examines whether the difference between $\theta_{O}$ and $\theta_{R}$ is smaller than a predefined equivalence threshold	Two null hypotheses are tested (using α=0.05) on whether the difference between $\theta_{O}$ and $\theta_{R}$ is larger or smaller than an equivalence threshold (i.e., ${\text{H}}_{0}:\theta_{O}-\theta_{R}\geq \delta_{E}$ and ${\text{H}}_{0}:\theta_{O}-\theta_{R}\leq -\delta_{E}$ where $\delta_{E}$ is the equivalence threshold). Replication success is concluded if both tests are statistically significant.

**Table 2:** Overview of the included methods for effect size estimation and testing the null hypothesis of no effect when combining one original study and one replication.

Method	Description
Fixed-effect (FE) model	Meta-analysis using the fixed-effect model
Bayesian fixed-effect model	Bayesian meta-analysis using the fixed-effect model with a normal distribution with mean zero and standard deviation 0.25 as prior for the effect size
Edgington	A hypothesis test based on the sum of *p*-values for testing that there is no effect in the original study and replication
Bayes factor replication	A Bayes factor based on only the data of the replication study. Bayes factors are computed using nonlocal alternative prior densities (Johnson, Pramanik, Shudde, 2023).
Hybrid method of meta-analysis	A method that meta-analyzes one original study and one replication by summing the *p*-values. It corrects for potential bias in the orginal study by taking statistical significance of the original study into account.
Extended hybrid method of meta-analysis (HYEMA)	A generalization of the hybrid method of meta-analysis that treats studies differently depending on whether studies are expected to be biased. In this paper, HYEMA was applied assuming a fixed-effect model and only the statistical significance of the original study was taken into account.
Snapshot hybrid	A Bayesian meta-analysis method for quantifying the evidence in favor for a zero, small, medium, or large effect by using posterior model probabilities, taking the statistical significance of the original study into account

The remainder of this paper is structured as follows. We continue with providing an overview of the different methods to assess replicability. In the next sections, we describe how we assessed the statistical properties of the methods by means of a numerical study and describe the properties of the methods. According to the results of this numerical study, all methods to assess the replicability have unfavorable properties. Hence, we focus on applying methods to meta-analyze the effect sizes of the original studies and replications when reanalyzing the SCORE data. We end this paper with a conclusion and discussion section.

### Methods to assess replicability

We focus on three aspects of replicability that are usually assessed in replicability projects: (1) is the original study successfully replicated, (2) what is the effect size estimate when combining the original study and replication and (3) is this effect different from zero. The included methods in our paper were all developed for analyzing one original study and one replication. There are also methods developed for Many Labs projects [e.g., 11], but these are not considered in this paper since the majority of original studies in the SCORE project were replicated once (259 out of 274, 94.5%).

### Assessing successful replication

Table 1 provides an overview of methods to assess whether a replication was successful and when a replication success is concluded in the analyses that are described later on in this paper. We have grouped the methods into two sets depending on the approach that is taken to determine successful replication. The first set of methods is based on assessing *the consistency between the conclusions of the original study and replication*. Within this set, the method “agreement in sign and statistical significance” is based on testing the null hypothesis of no effect in the original study and replication. Replication success is concluded with this method if there is simultaneous agreement in (i) statistical significance of the original study and replication, and (ii) the direction of the effect size estimate in both studies [e.g., 32, 36]. This method is frequently applied and included in replicability projects [3–5].

The “sceptical *p*-value” [20,37] uses the data of the original study to create a prior such that the lower bound of the credible interval of the posterior distribution equals zero. Subsequently, there is replication success if this prior distribution is in conflict with the data of the replication based on a frequentist hypothesis test. The “replication Bayes factor” [19,38] examines whether $\theta_{R}$, which is the true effect size of the replication, is more likely under a model with a true effect of zero compared with a model where $\theta_{R}$ follows the posterior distribution of the original study using the Bayes factor. More evidence in favor of the model that equals the posterior distribution of the original study is indicative for replication success.

The methods in the second set of Table 1 assess *the similarity of both studies’ true effect sizes* $\theta_{O}$ *and* $\theta_{R}$. The first three methods are testing ${\text{H}}_{0}:\theta_{O}=\theta_{R}$ by evaluating whether ${\hat{\theta }}_{O}$ is in the 95% CI for $\theta_{R}$ and ${\hat{\theta }}_{R}$ is in the 95% PI for $\theta_{O}$ [16]. The hypothesis ${\text{H}}_{0}:\theta_{O}=\theta_{R}$ can also be tested with the Cochran’s *Q*-test, which is frequently used in meta-analysis to test for homogeneity in true effect size [39]. The *Q*-test can be used for testing *exact* and *approximate* replication [25]. Exact replication implies testing ${\text{H}}_{0}:\theta_{O}=\theta_{R}$. Approximate replication allows for small differences between $\theta_{O}$ and $\theta_{R}$ that the researcher can define (for guidance on defining a small difference see Hedges et al. [25]). Schauer et al. [23] noted that the method based on the 95% PI and the *Q*-test for exact replication are equivalent in case of one original study and one replication. Moreover, the 95% PI and the *Q*-test are the uniformly most powerful unbiased tests [21] implying that the 95% PI for $\theta_{O}$ and the *Q*-test always have higher statistical power than other unbiased tests of the same hypothesis if the model assumptions are met.

Another method for assessing similarity between $\theta_{O}$ and $\theta_{R}$ is the correspondence test [18]. This test combines the results of two hypothesis tests: a difference and equivalence test. In the difference test, the null hypothesis of equal true effect size in the original study and replication (i.e., ${\text{H}}_{0}:\theta_{O}=\theta_{R}$) is tested. In the equivalence test, two null hypotheses are tested on whether the difference between the true effects is larger or smaller than an equivalence threshold (i.e., ${\text{H}}_{0}:\theta_{O}-\theta_{R}\geq \delta_{E}$ and ${\text{H}}_{0}:\theta_{O}-\theta_{R}\leq -\delta_{E}$ where $\delta_{E}$ is the equivalence threshold). Equivalence is concluded if both tests are statistically significant, because this implies that the difference between $\theta_{O}$ and $\theta_{R}$ is smaller than $\delta_{E}$ and larger than $-\delta_{E}$ (i.e., $|\theta_{O}-\theta_{R}|<\delta_{E}$). The correspondence test yields four different conclusions depending on statistical significance of the difference and equivalence test (i.e., equivalent, trivially different, different, and indeterminate). In SCORE [32], equivalence was considered to be a replication success regardless of the result of the difference test. In SCORE [32], replication success was concluded if equivalence was established based on the equivalence test alone (i.e., regardless of the outcome of the difference test), meaning that it was concluded that $|\theta_{O}-\theta_{R}|<\delta_{E}$. Hence, we include the equivalence test instead of the correspondence test in the analyses of this paper.

In SCORE [32], a replication success was also concluded if ${\hat{\theta }}_{R}$ was in the 95% CI for $\theta_{O}$. We excluded this method because the replication typically had larger sample size than the original study, leading to a smaller standard error and hence a narrower CI and more powerful hypothesis test. The small telescopes method [17] was also included in SCORE [32] and tests whether $\theta_{R}$ is equal to or larger than an effect size with 33% statistical power based on the sample size of the original study. We did not include this method in our paper, because it is not about the (in)consistency between the conclusions of the original study and replication or the (dis)similarity of $\theta_{O}$ and $\theta_{R}$.

### Estimating effect size and testing for no effect

We also included seven meta-analysis methods to estimate the meta-analytic effect size and/or test the null hypothesis of no meta-analytic effect when combining an original study and a replication. All included methods assume that the same true effect size is estimated in the original study and replication. The included methods are summarized in Table 2. The first four methods listed in Table 2 were also implemented in SCORE [32], that is, frequentist and Bayesian FE model, Edgington’s method, and a Bayes factor using only the data of the replication. In contrast to SCORE, we focus on hypothesis testing of the meta-analytic effect size *and* effect size estimation.

The first two methods are the FE model [30] implemented in a frequentist as well as in a Bayesian framework. In these models, the meta-analytic estimate is computed under the assumption that estimates of $\theta_{O}$ and $\theta_{R}$ are unbiased. The third method in Table 2, Edgington’s method [15], is a method that does not yield a meta-analytic estimate. The sum of the *p*-values is computed in this method for testing the null hypothesis of no effect in the original study and replication. The Bayes factor replication method is a Bayesian hypothesis test based on only the data of the replication that tests a two-sided hypothesis on whether the effect is equal to zero. In SCORE [32], a Bayes factor using nonlocal alternative densities was implemented [40]. The noncentrality parameter of the prior distribution is repeatedly changed in this testing procedure, which results in a range of Bayes factors. The reported Bayes factor in SCORE is the largest Bayes factor in this range.

The last three methods listed in Table 2, hybrid method of meta-analysis, extended hybrid method of meta-analysis, and snapshot hybrid, were not included in SCORE. The key characteristic of these methods is that only the effect size estimator of $\theta_{R}$ is assumed to be unbiased. By taking into account that the original effect size is statistically significant (i.e., conditioning on statistical significance), the methods correct for potential bias in $\theta_{O}$. The hybrid method of meta-analysis (abbreviated as hybrid [13],) estimates the meta-analytic effect size and tests the null hypothesis of no effect. Hybrid and Edgington’s method both use the *p*-values for testing the null hypothesis of no effect, but hybrid corrects for potential selection bias in the original study. The extended hybrid method of meta-analysis (abbreviated as HYEMA [41],) is a generalization of hybrid that allows for the inclusion of more than one original study and one replication and allows for estimating the between-study variance in true effect size. HYEMA can, however, also be applied to the case with only one original study and one replication and when the between-study variance is assumed to be zero (i.e., FE model). Another difference between hybrid and HYEMA is that hybrid uses the sum of *p*-values for effect size estimation and statistical inference and HYEMA is implemented in a maximum likelihood framework. The last method in Table 2, the snapshot hybrid method [14] applies a Bayesian framework to compute posterior model probabilities (PMP) that quantify the evidence in favor of a zero, small, medium, and large effect size, using Cohen’s rules of thumb [42]. Hence, the method quantifies how likely it is that the effect size has a particular magnitude rather than providing a point estimate.

In the next section of the paper, we study the statistical properties of the methods by means of a numerical study. This numerical study is tailored to the data observed in SCORE. Based on these statistical properties, methods will be selected that are applied to the SCORE data later in the paper.

## Evaluation of the statistical properties of the methods

### Methods

We evaluated the statistical properties of the included methods by employing a similar procedure as in van Aert and van Assen (2017, 2018). We tailored the evaluation of the statistical methods to the 225 original studies and replications in the SCORE project for which effect sizes were transformed to correlation coefficients. We did not analyze correlation coefficients directly but first applied the Fisher’s *z* transformation to make sure that the sampling variance is independent of the correlation [43] and because the sampling variance of the transformed correlations more closely follows the normal distribution. This is a desirable property given that many of the included methods assume a normally distributed effect size measure.

A large portion of the original studies were reported as statistically significant in the SCORE project when using a two-tailed test with α=0.05 (216 out of 225, 96.0%). Hence, we assumed a statistically significant original study by conditioning on the statistical significance of the effect size of this study. Without loss of generality, we assumed the original effect was positive. The conditional probability density function of the original study was obtained by first computing the statistical power π of observing a statistically significant effect size when testing a right-tailed hypothesis with α=0.025 (which is equivalent to two-tailed testing with α=0.05 but focusing on one direction) given the true effect size $\theta_{O}$ and variance $\sigma_{O}^{2}$. The probability π was computed using the cumulative distribution function of the normal distribution. Subsequently, 100 equally distributed cumulative probabilities $P_{i}^{O}$ were created using


$$\begin{array}{c} P_{i}^{O}=1-\pi +\frac{i\times \pi }{101} \end{array}$$

where i=1,\hdots,100. The $P_{i}^{O}$ were transformed into Fisher’s *z* correlations of the original study using the quantile function of the normal distribution with mean $\theta_{O}$ and variance $\sigma_{O}^{2}$ in order to approximate the marginal probability density function.

Effect sizes of the replication were generated in an analogous way as for the original study. The difference between generating data for the two types of studies was that there was no requirement for the replication to be statistically significant. That is, the marginal probability density function of the replication was obtained by creating 100 equally distributed cumulative probabilities $P_{i}^{R}$ using


$$\begin{array}{c} P_{i}^{R}=\frac{i}{101}. \end{array}$$

Corresponding Fisher’s *z* transformed correlations were computed using the quantile function of the normal distribution with mean $\theta_{R}$ and variance $\sigma_{R}^{2}$.

The joint distribution of the statistically significant original study and replication was approximated by combining all possible Fisher’s *z* transformed correlations of the original study and replication. This resulted in a grid of 100 × 100 = 10,000 combinations to which the methods evaluated in this study were applied. This procedure yields numerical approximations of the expectations of the outcome measures described below.

Conditions for $\theta_{O}$, $\theta_{R}$, $\sigma_{O}^{2}$, and $\sigma_{R}^{2}$ were selected in such a way to closely match the characteristics of the data in the SCORE project. The following Pearson correlation coefficients of the original study ($\rho_{O}$) and replication ($\rho_{R}$) were selected: 0, 0.1, 0.3, and 0.5. These values resembled a zero, small, medium, and large effect according to the rules-of-thumb by Cohen [42]. These Pearson correlation coefficients implied the following Fisher’s *z* transformed correlations of the original study ($\theta_{O}$) and replication ($\theta_{R}$): 0, 0.100, 0.310, and 0.549. The zero, small, and medium effects were also close to the observed Fisher’s *z* transformed correlations observed in the replications of the SCORE project (first quartile 0.049, median 0.134, and third quartile 0.252).

We included conditions were $\theta_{O}$ and $\theta_{R}$ were equal to each other to examine whether the methods could detect replication success. We also included conditions where $\theta_{O}$ was larger than $\theta_{R}$ in order to study replication failure. In these conditions, $\theta_{O}$ was always one magnitude of the rules-of-thumb by Cohen [42] larger than $\theta_{R}$. That is, $\theta_{O}=0.100$ was combined with $\theta_{R}=0$, $\theta_{O}=0.310$ was combined with $\theta_{R}=0.100$, and $\theta_{O}=0.549$ was combined with $\theta_{R}=0.310$.

Conditions for $\sigma_{O}^{2}$ and $\sigma_{R}^{2}$ were computed with the estimation equation of the variance of Fisher’s *z* correlations, which is 1/(N−3) where *N* is the sample size of the original study or replication. This sample size was set equal to the first quartile, median, and third quartile of the sample sizes observed in the original studies (median *N* = 218, first quartile = 97 and third quartile = 2,236) and replications (median *N* = 556, first quartile = 215 and third quartile = 2,231) in the SCORE project. We also added a sample size of 10,000 for the replication to study the large sample statistical properties of the methods. This yielded the following conditions for $\sigma_{O}^{2}$ = (0.0106, 0.0047, 0.00045) and for $\sigma_{R}^{2}$ = (0.0047, 0.0018, 0.00045, 0.00010).

The methods listed in Table 1 were included to study replication success. The last column of Table 1 details the criterion of each method for concluding replication success. These criteria were adopted from the SCORE project. The outcome of these methods was the replication success probability that was computed by dividing the cases were replication success was concluded by 10,000. The methods listed in Table 2 were included for testing the null hypothesis of no effect and effect size estimation. The outcomes for hypothesis testing were Type-1 error rate and statistical power. That is, how often the null hypothesis was rejected in the absence (i.e., Type-1 error rate) and presence of an effect (i.e., statistical power). For effect size estimation, the outcomes were bias and Root Mean Square Error (RMSE). The median of the posterior distribution was used as effect size estimate for the Bayesian FE model. Fisher’s *z* transformed correlations were analyzed with all included methods. Estimates of the Fisher’s *z* transformed correlations were (back-)transformed to correlation coefficients (using equation (11.39) in [44]) to facilitate interpretation of bias and RMSE of the effect size estimators. Note that we assume selection of originally statistically significant findings, hence all reported outcome measures are conditional on the original study being statistically significant by design of the numerical study.

We used α=0.05 for methods that tested hypotheses in a frequentist framework except for the sceptical *p*-value where we used the default “golden” implementation implying α = 0.123 [37]. For the methods based on Bayes factors, we implemented multiple thresholds, because different thresholds were used in the SCORE project. A successful replication was concluded in the SCORE project for a Bayes factors larger than 1 for the replication Bayes factor, larger than 3 for the Bayesian FE model, and larger than 10 for the Bayes factor based on the replication study. We implemented these thresholds as well as 20 and 150 for the methods based on Bayes factors, because these are commonly used thresholds in practice [45].

For the snapshot hybrid method, we examined how often the PMP was largest for the true effect size and how often it exceeded 0.75, indicating cases where there was more than three times as much evidence for the true model as for the alternatives. A PMP larger than 0.75 is in line with the Bayes factor threshold 3, because it indicates that there was three times more evidence for one model compared to another model. We also studied as outcome the coverage probability of the frequentist FE model, PI, hybrid, and HYEMA.

This analysis was programmed in R [46, Version 4.4.3]. We used the R packages “BFF” [47, Version 4.5.0], “hypergeo” [48, Version 1.2.14], “metaBMA” [49, Version 0.6.9], “puniform” [50, Version 0.2.9], and “ReplicationSuccess” [20, Version 1.3.3]. R code of our analyses is available at https://osf.io/acbd5.

## Results

We report the statistical properties separately for the methods that focus on assessing successful replication and estimating the effect size and testing the null hypothesis of no effect. For all Bayesian methods, we report the results for concluding successful replication or rejecting the null hypothesis of no effect if the Bayes factor in favor of this hypothesis was larger than 3. Results of Bayes factor methods based on a different threshold than 3, testing for approximate replication with the *Q*-test, and the coverage probability are not reported in this section and are available in the supplemental materials (https://osf.io/ktazu).

### Assessing successful replication

Fig 1 shows the results of the methods examining replication success if the two studies’ underlying true effect sizes are different. The subfigures show the probability of replication success (*y*-axis) for different combinations of sample sizes of the original study (varied in the columns) and replication (varied in the rows). In this figure, the true effect size (*x*-axis) of the original study was always one magnitude of the rules-of-thumb by Cohen [42] larger than that of the replication.

**Fig 1:**
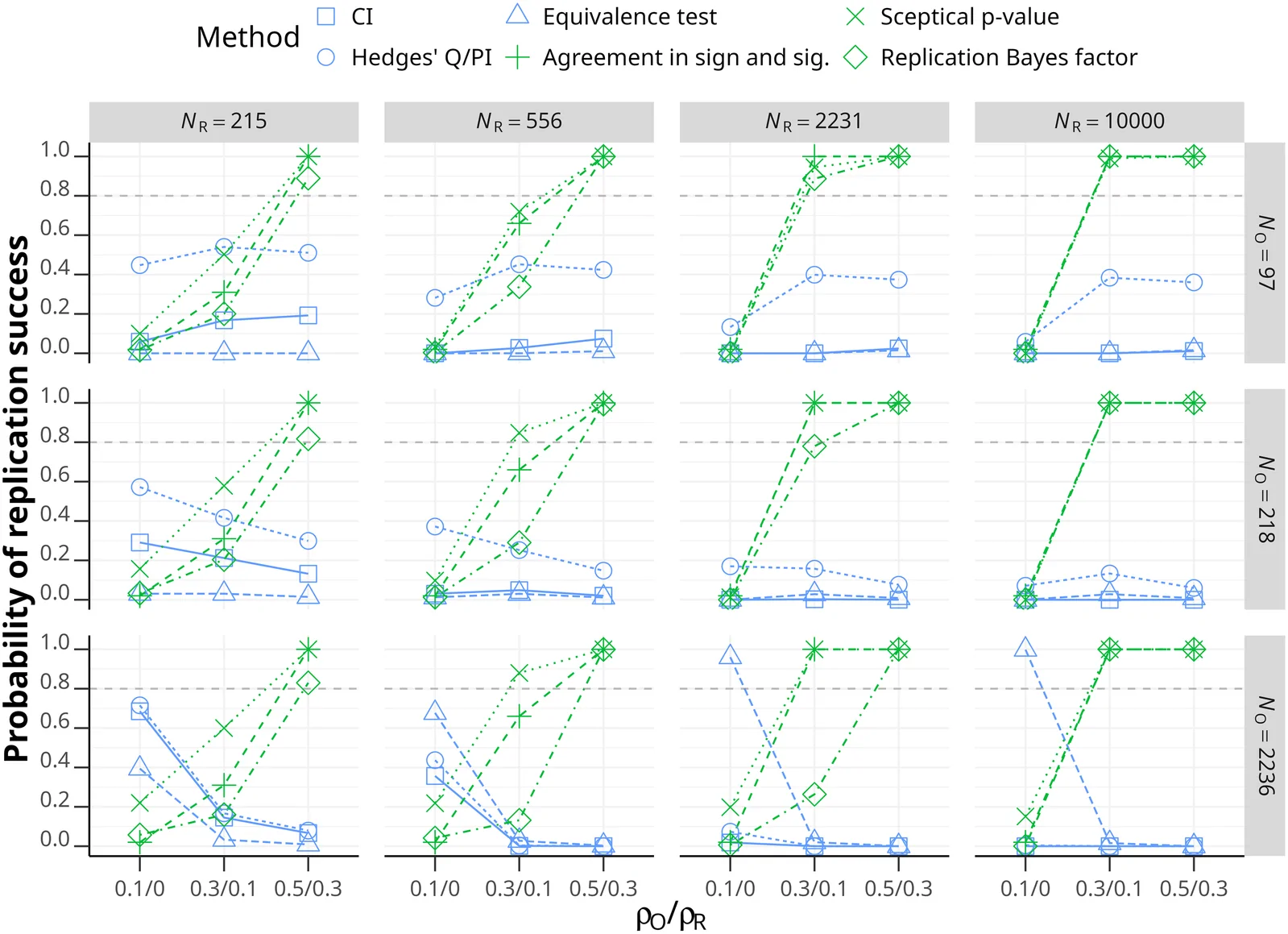
Probability of replication success if the true effect sizes of the original study and replication are different. The included methods are whether the original study’s effect size is in the 95% confidence interval (CI) of the replication, equivalence test, Sceptical *p*-value, Hedges’ *Q*/95% prediction interval (PI), agreement in sign and statistical significance, and replication Bayes factor. The numbers on the right-hand side and on the top of the figure refer to the sample sizes of the original study and replication, respectively. The *x*-axis refers to the true Pearson correlation of the original study ($\rho_{O}$) and replication ($\rho_{R}$).

Considering first the methods that assess consistency of both studies’ conclusions (colored green in the figure), optimal performance is as follows. If $\rho_{O}$=0.1/$\rho_{R}$=0, ideally the probability of replication success equals 0 for the replication Bayes factor, 0.123 for the sceptical *p*-value, and 0.025 (i.e., half of α) for agreement in sign and statistical significance. If both true effect sizes are different but larger than 0, the optimal probability of replication success is 1.

To summarize the results of these methods, we first turn to the conditions with $N_{R}$ = 10,000. The replication Bayes factor performed optimal, whereas the sceptical *p*-value showed suboptimal performance for $\rho_{O}$=0.1/$\rho_{R}$=0 (probability 0 for $N_{O}$ = 97 and 218). For $\rho_{O}$=0.1/$\rho_{R}$=0, the probability of replication success was close to ideal with on average 0.020 for agreement in sign and statistical significance. Turning to conditions with $N_{R}$ < 10,000, we focus first on the condition $\rho_{O}$=0.1/$\rho_{R}$=0. Here, the replication Bayes factor and agreement in sign and statistical significance performed well with high probabilities (> 0.95; i.e., 1 – replication success probability) that both studies’ conclusions are different. The sceptical *p*-value performed worse but reasonably well with probabilities close to 0.8 (the worst performance is for $N_{R}$ = 215, $N_{O}$ = 2,236, namely 0.780).

The results for the other conditions ($\rho_{O}$=0.3/$\rho_{R}$=0.1 and $\rho_{O}$=0.5/$\rho_{R}$=0.3) were mostly determined by the sample size of the replication. This means that the results within one column of subfigures with the same $N_{R}$ were largely similar. If $\rho_{R}$=0.3 all three methods provided correct conclusions with probability larger than 0.8 for $N_{R}\geq$ 215, with close to optimal performance for $N_{R}$ ≥ 556, reflecting the larger sample size and thereby larger statistical power for $\rho_{R}$=0.3. Focusing on $\rho_{R}$=0.1, a larger sample size of the replication was required to have high replication success probabilities, with good performance of agreement in sign and statistical significance and the sceptical *p*-value for $N_{R}$ = 2,231. The replication Bayes factor did not perform well in case of $\rho_{R}$ = 0.1 if the original sample size was large ($N_{O}$ = 2,236), because the replication with $\rho_{R}$=0.1 was more in line with the model of a null effect than with the model where the effect size equaled the original study’s observed effect size. For a smaller sample size of the replication (i.e., $N_{R}$ = 556), only the sceptical *p*-value showed reasonable to good performance (probability ≥ 0.782) when $\rho_{R}$=0.1. Also generally, the sceptical *p*-value performed best of the three methods in conditions where the true effect sizes were different between original study and replication.

Considering the methods that assess similarity of $\rho_{O}$ and $\rho_{R}$ (colored blue), optimal performance is a probability of replication success equal to 0 as the two studies’ true effect sizes are unequal, and a probability of 1 for the equivalence test if $\rho_{O}$=0.1/$\rho_{R}$=0, as the difference between the two effect sizes is smaller than the equivalence threshold (0.2). Only if both studies’ sample sizes were large ($N_{O}$ = 2,236, $N_{R}\geq$ 2,231) all three methods (Hedges’ *Q*/PI, CI, equivalence test) performed well. Lower original study or replication sample sizes negatively affected performance. For Hedges’ *Q*/PI and the equivalence test, one large study’s sample size cannot compensate for the other study’s small sample size. For instance, performance for Hedges’ *Q*/PI was 0.4 or lower if $N_{O}$ = 97 and $N_{R}$ = 10,000. The equivalence test did not detect ‘equivalence’ of $\rho_{O}$=0.1/$\rho_{R}$=0 well for ($N_{O}$ = 2,236, $N_{R}$ = 556). This is because the standard error used in both tests are a function of both studies’ sampling error. Hence, we recommend not using these methods when evaluating replication success when one of the study sample sizes is not very large. On the other hand, the performance of the CI was mostly dependent on the replication sample size. The CI showed good performance (probability ≤ 0.001) for $N_{R}\geq$ 556, independent of the original study’s sample size, except when both true effect sizes were close together ($\rho_{O}$=0.1/$\rho_{R}$=0).

Fig 2 shows the replication success probability if the true effect size of the original study and replication is the same. We again first consider methods assessing the consistency between the conclusions of the original study and replication (green color). For a true effect size equal to zero, we expect replication success probabilities that are close to zero. Optimal performance equals 1 for the nonzero true effect sizes. Turning to the results, as statistical power of the test of the null hypothesis increases in true effect size and sample sizes, the three methods’ probabilities of replication success increased as a function of these parameters. For effect sizes 0.3 and 0.5 performance was good (probabilities equaled 1), irrespective of both studies’ sample sizes. As the original effect size was statistically significant, both studies’ conclusions were only consistent if the sample size of the replication was large enough. For a true effect size of 0.1, a replication sample size of $N_{R}\geq$ 2,231 was needed for good performance. For a zero true effect size, agreement in sign and statistical significance and the replication Bayes factor showed low probabilities (< 0.048), irrespective of both studies’ sample sizes. The sceptical *p*-value also showed low probabilities (≤ 0.208) but larger than the other two methods in case of a zero true effect size. However, keep in mind that for a zero true effect size the studies’ conclusions are different, but the underlying effect sizes are the same.

**Fig 2:**
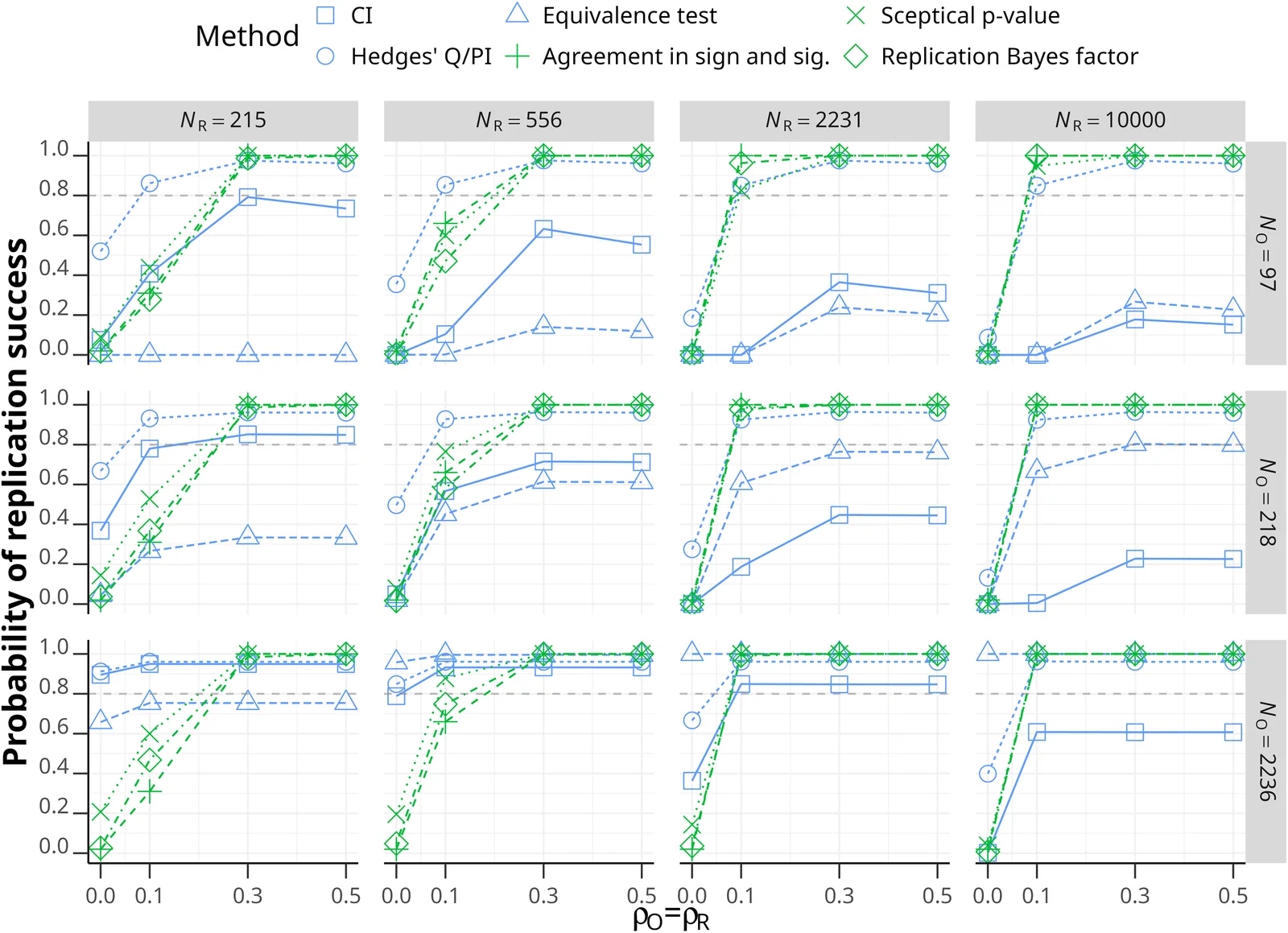
Probability of replication success if the true effect sizes of the original study and replication are the same. The included methods are whether the original study’s effect size is in the 95% confidence interval (CI) of the replication, equivalence test, Sceptical *p*-value, Hedges’ *Q*/95% prediction interval (PI), agreement in sign and statistical significance, and Replication Bayes factor. The numbers on the right-hand side and on the top of the figure refer to the sample sizes of the original study and replication, respectively. The *x*-axis refers to the true Pearson correlation of the original study ($\rho_{O}$) and replication ($\rho_{R}$).

The optimal performance of the methods assessing similarity of true effect sizes $\rho_{O}$ and $\rho_{R}$ (colored blue) was a probability of replication success equal to 1, for all true effect sizes. But, here again, low replication success probabilities were expected under a zero true effect size, as the original effect size was statistically significant. Discussing the three methods one by one, the equivalence test performed well (probabilities ≥ 0.958) if both studies’ sample sizes were large ($N_{O}$ = 2,236 and $N_{R}\geq$ 556). Performance particularly decreased for small sample sizes of the original study ($N_{O}$ = 97; probability equaled zero for $N_{R}$=215), because the overestimation was the largest for this condition due to conditioning on statistical significance. The performance of Hedges’ *Q*/PI was good (probabilities close to 0.95, corresponding to 1 – Type I error of the test) in all conditions where the bias of the original study’s effect size estimator was minimal. This bias was substantial for a zero true effect size, leading to generally bad performance particularly when the replication sample size was large. Finally, the performance of the CI was bad in many conditions, for obvious reasons; if the sample size of the replication was larger than of the original study (which is also often the case in practice), then the CI was relatively narrow and will infrequently contain the original effect size because that will fluctuate more. This issue was aggravated at zero true effect size due to the bias of the original study’s effect size estimator. Hence, the CI should not be used to assess replication success.

To summarize, assessing successful replication was challenging if the true effect size was zero or small and sample sizes of the original study and replication were not very large. The performance of all methods improved when the sample size of the original study increased, because the larger sample size diminished the bias in the original study’s effect size estimator. These results show that there was often not enough information in case of one original study and one replication to reliably assess replication success. For this reason, we do not recommend applying methods to assess consistency between the conclusion of the original study and replication as these showed especially unfavorable properties in case of small sample sizes. For methods assessing similarity of $\rho_{O}$ and $\rho_{R}$, we showed that methods perform well when (i) both studies’ sample sizes were large, and (ii) the true effect was nonzero. Condition (i) often does not apply in practice, and (ii) is practically very relevant. As the equivalence test with a small threshold (i.e., 0.2) also performed well for a zero true effect size, we only recommend this test when both study sample sizes are large.

### Estimating effect size and testing for no effect

Table 3 shows the bias of the estimators of frequentist and Bayesian FE model, hybrid, and HYEMA. A bold number in the table indicates that this method had the smallest bias in a particular condition. HYEMA and hybrid were the least biased if the original study was most biased. That is, these methods had the least bias for a zero and small true effect size in combination with small and moderate sample sizes. For the other conditions, HYEMA and hybrid also had only a small absolute bias (never exceeding 0.01). The frequentist FE model was positively biased. Its bias was especially substantial if the true effect size was zero (maximum bias 0.080). This bias decreased when the true effect size and sample sizes increased, and the frequentist FE model was the least biased method in these conditions. The Bayesian FE model was never the least biased method (bias was rarely below 0.01). This method had a positive bias for no and a small true effect due to the biased original study and a negative bias for a medium and large true effect. This was caused by the relatively informative prior centered at zero that shrunk the estimate towards zero. Comparing and interpreting all methods’ performances, HYEMA typically performed best as its estimator was close to unbiased and also the estimator based on the frequentist FE model had a small amount of bias if the statistical power was close to 1.

**Table 3:** Bias of the fixed-effect model (FE), the Bayesian fixed-effect model (Bayes), the hybrid method of meta-analysis (Hybrid), and the hybrid extended meta-analysis (HYEMA). Numbers in bold indicate that this method had the lowest bias in a particular condition.

$N_{O}$	$N_{R}$	ρ=0				ρ=0.1				ρ=0.3				ρ=0.5			
		FE	Bayes	Hybrid	HYEMA	FE	Bayes	Hybrid	HYEMA	FE	Bayes	Hybrid	HYEMA	FE	Bayes	Hybrid	HYEMA
97	215	0.074	0.070	−0.003	**−0.001**	0.047	0.038	−0.004	**−0.002**	0.007	−0.016	−0.004	**−0.003**	**−0.001**	−0.056	−0.001	−0.001
	556	0.035	0.034	−0.001	**−0.000**	0.022	0.019	−0.002	**−0.000**	0.003	−0.012	−0.002	**−0.001**	**−0.001**	−0.046	−0.001	−0.001
	2,231	0.010	0.010	−0.000	**−0.000**	0.006	0.005	−0.000	**−0.000**	0.001	−0.010	−0.000	**−0.000**	**−0.000**	−0.040	−0.000	−0.000
	10,000	0.002	0.002	−0.000	**−0.000**	0.001	0.001	−0.000	**−0.000**	0.000	−0.009	−0.000	**−0.000**	**−0.000**	−0.038	−0.000	−0.000
218	215	0.080	0.077	−0.005	**−0.002**	0.038	0.032	−0.005	**−0.004**	**−0.000**	−0.019	−0.001	−0.001	−0.001	−0.051	**−0.001**	−0.001
	556	0.044	0.043	−0.002	**−0.000**	0.021	0.018	−0.002	**−0.001**	**−0.000**	−0.014	−0.001	−0.001	**−0.000**	−0.045	−0.000	−0.000
	2,231	0.014	0.014	−0.001	**−0.000**	0.007	0.006	−0.001	**−0.000**	**−0.000**	−0.010	−0.000	−0.000	**−0.000**	−0.040	−0.000	−0.000
	10,000	0.003	0.003	−0.000	**−0.000**	0.002	0.001	−0.000	**−0.000**	**−0.000**	−0.009	−0.000	−0.000	**−0.000**	−0.038	−0.000	−0.000
2,236	215	0.045	0.045	**−0.009**	−0.011	**0.000**	−0.001	−0.001	−0.001	−0.000	−0.011	−0.000	**−0.000**	**−0.000**	−0.040	−0.000	**−0.000**
	556	0.040	0.039	−0.005	**−0.004**	**0.000**	−0.001	−0.000	−0.001	**−0.000**	−0.010	−0.000	−0.000	**−0.000**	−0.040	−0.000	−0.000
	2,231	0.025	0.025	−0.001	**−0.001**	**0.000**	−0.001	−0.000	−0.000	−0.000	−0.010	**−0.000**	−0.000	**−0.000**	−0.039	−0.000	**−0.000**
	10,000	0.009	0.009	−0.000	**−0.000**	**0.000**	−0.000	−0.000	−0.000	**−0.000**	−0.009	−0.000	−0.000	**−0.000**	−0.038	−0.000	**−0.000**

Bias is shown for Pearson correlation coefficient (ρ) as effect size measure.

Table 4 shows the RMSE of the different methods where bold numbers indicate the method that has the lowest RMSE for a particular condition. For interpreting the results, note that RMSE equals squared bias plus squared standard error. For a zero true effect size, HYEMA generally had the lowest RMSE followed by hybrid, and the Bayesian and frequentist FE model, because HYEMA and hybrid were close to unbiased. As bias of the other methods decreased in the true effect size, differences in RMSE between all methods decreased as a function of the true effect size. For a small true effect size, the Bayesian FE model outperformed the other methods, because of the small positive bias in combination with the relatively informative prior distribution centered at zero. For medium and large true effect sizes, the frequentist FE model and HYEMA showed the best and comparable performance, with the frequentist FE model slightly outperforming HYEMA due to the small bias coupled with high precision in these conditions. The Bayesian FE model generally performed worst, with hybrid only approaching best performance when both studies’ sample sizes were large.

**Table 4:** Root mean square error (RMSE) of the fixed-effect model (FE), the Bayesian fixed-effect model (Bayes), the hybrid method of meta-analysis (Hybrid), and the hybrid extended meta-analysis (HYEMA). Numbers in bold indicate that this method had the lowest RMSE in a particular condition.

$N_{O}$	$N_{R}$	ρ=0				ρ=0.1				ρ=0.3				ρ=0.5			
		FE	Bayes	Hybrid	HYEMA	FE	Bayes	Hybrid	HYEMA	FE	Bayes	Hybrid	HYEMA	FE	Bayes	Hybrid	HYEMA
97	215	0.087	0.082	0.077	**0.064**	0.066	**0.058**	0.073	0.062	0.047	**0.044**	0.057	0.053	**0.041**	0.064	0.042	0.041
	556	0.050	0.048	0.051	**0.040**	0.041	**0.038**	0.049	0.040	0.034	**0.032**	0.040	0.035	**0.028**	0.052	0.031	0.028
	2,231	0.022	0.022	0.027	**0.020**	0.020	**0.020**	0.026	0.020	**0.018**	0.019	0.023	0.018	**0.015**	0.042	0.018	0.015
	10,000	0.010	0.010	0.013	**0.010**	0.010	**0.009**	0.013	0.010	**0.009**	0.012	0.012	0.009	**0.007**	0.039	0.009	0.007
218	215	0.087	0.083	0.071	**0.061**	0.052	**0.047**	0.063	0.057	0.042	**0.042**	0.043	0.044	**0.035**	0.058	0.035	0.035
	556	0.054	0.052	0.048	**0.040**	0.037	**0.034**	0.044	0.038	**0.031**	0.032	0.033	0.032	**0.026**	0.050	0.027	0.026
	2,231	0.023	0.023	0.026	**0.020**	0.020	**0.019**	0.025	0.020	**0.018**	0.019	0.020	0.018	**0.015**	0.042	0.016	0.015
	10,000	0.010	0.010	0.013	**0.010**	0.009	**0.009**	0.013	0.009	**0.009**	0.012	0.011	0.009	**0.007**	0.039	0.009	0.007
2,236	215	0.046	**0.045**	0.051	0.046	0.019	**0.019**	0.023	0.021	0.018	0.019	0.020	**0.018**	**0.015**	0.042	0.016	**0.015**
	556	0.041	0.040	0.037	**0.033**	0.018	**0.018**	0.020	0.019	**0.017**	0.018	0.017	0.017	0.014	0.041	0.014	**0.014**
	2,231	0.027	0.027	0.022	**0.019**	0.014	**0.014**	0.014	0.015	0.013	0.015	**0.013**	0.013	0.011	0.040	**0.011**	0.011
	10,000	0.012	0.012	0.012	**0.009**	0.009	**0.009**	0.009	0.009	**0.008**	0.012	0.008	0.008	**0.007**	0.039	0.007	0.007

RMSE is shown for Pearson correlation coefficient (ρ) as effect size measure.

Fig 3 shows the Type-1 error rate and statistical power of the methods to test the null hypothesis of no effect. The deviation from the nominal Type-1 error rate was the largest in the condition with the smallest sample size of the replication (i.e., $N_{R}=215$) for the frequentist and Bayesian FE model (maximum Type-1 error rates 0.728 and 0.156 for frequentist and Bayesian). The Type-1 error rate of Edgington’s method (0.018), the Bayes factor based on only the replication (0.040), and hybrid (0.046) were constant across all conditions. HYEMA’s Type-1 error rate (0.040) deviated the most from the nominal rate for the condition $N_{O}$ = 2,236 and $N_{R}$ = 10,000. Hence, the Bayes factor based on only the replication, hybrid, and HYEMA yielded the Type-1 error rates that were closest to the nominal rate of α=0.05.

**Fig 3:**
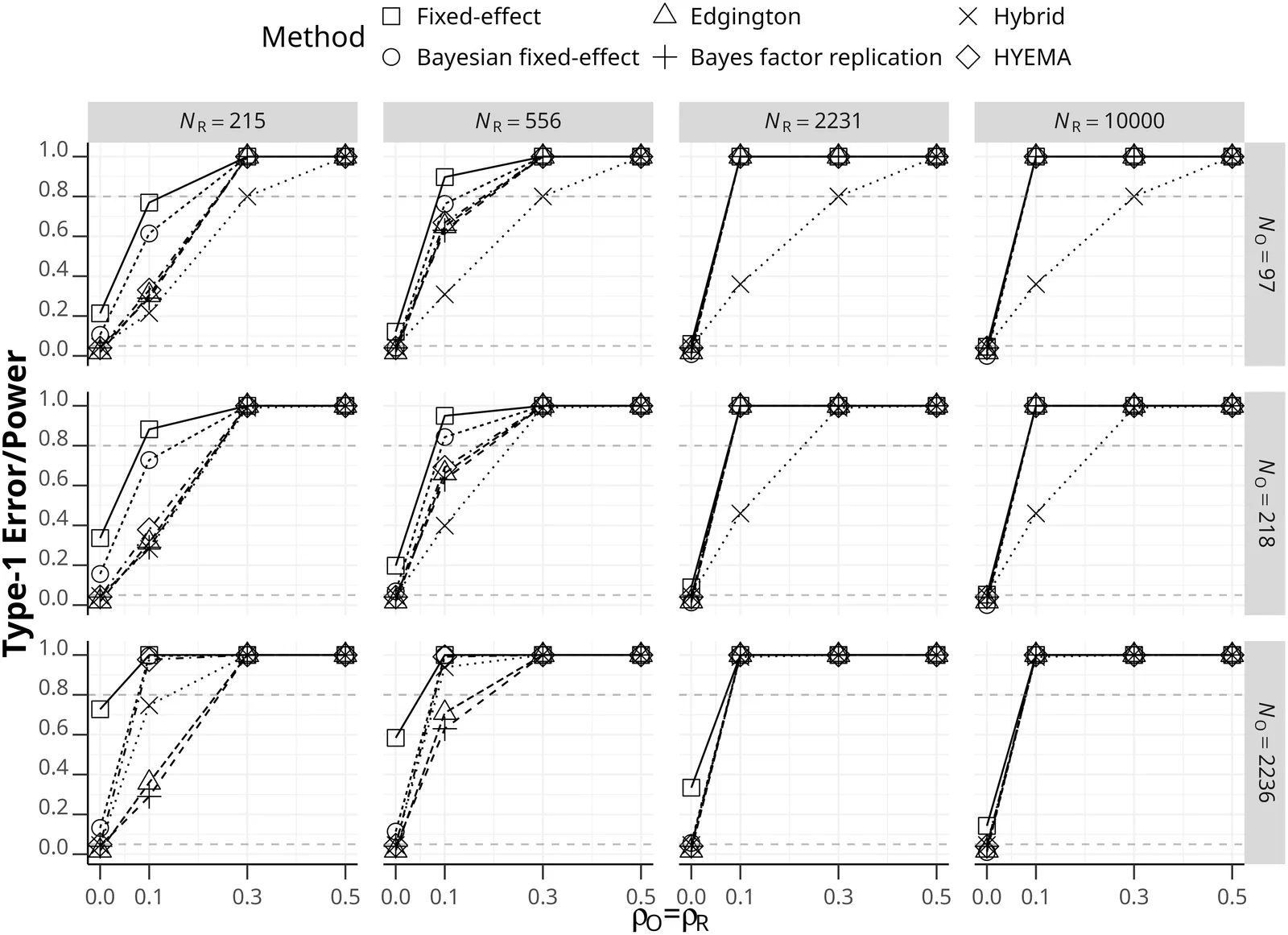
Type-1 error rate (for correlations equal to zero) and statistical power (for all other correlations) of the fixed-effect model, Bayesian fixed-effect model, Edgington, Bayes factor based on only the data of the replication (Bayes factor replication), hybrid method of meta-analysis (Hybrid), and hybrid extended meta-analysis method (HYEMA). The numbers on the right-hand side and on the top of the figure refer to the sample sizes of the original study and replication, respectively. The *x*-axis refers to the true Pearson correlation of the original study ($\rho_{O}$) and replication ($\rho_{R}$).

The deviations from the nominal Type-1 error rate made interpreting the results of the methods’ statistical power in Fig 3 complicated. The frequentist and the Bayesian FE model had the largest statistical power, but this high power was partially caused by the inflated Type-1 error rate. All methods had statistical power larger than 0.8 if the true effect size was 0.3 or larger. For a true effect size of 0.1, methods only passed the 0.8 threshold if the sample size of the original study and/or replication was sufficiently large. A clear finding was that the statistical power of hybrid was lower than of the other methods, particular if the original sample size was small. This again illustrated lower precision of hybrid compared to the other methods. Of all methods with appropriate Type-1 error rate (i.e., Edgington, Bayes factor based on only the replication data, hybrid, and HYEMA), HYEMA had the highest statistical power in all conditions, with only insufficient statistical power (< .8) for small true effect size if $N_{O}\leq$ 218 in combination with $N_{R}\leq$ 215. That is, when the sum of both studies’ sample sizes was not large.

Table 5 shows the average PMP (first four columns) and proportion of times the PMP was larger than 0.75 (last four columns) of the snapshot hybrid method. The results are shown for the cases where the effect size assumed in the snapshot was equal to the true effect size. Performance of the snapshot method was good (average PMP as well as percentage PMP larger than 0.75) for true effect sizes 0.3 and 0.5, and when $N_{R}\geq$ 556 for true effect sizes 0 and 0.1. Clearly, performance increased as a function of the true effect size and sample sizes. The lowest average PMP was 0.629 for a true effect size of 0.1 and small sample sizes in the original study ($N_{O}=97$) and replication ($N_{R}=215$).

**Table 5:** Average posterior model probability (PMP) and percentage of times the PMP was larger than 0.75 of the snapshot hybrid method. The results are shown for the effect size of the snapshot that is equal to the true effect size in the numerical study.

$N_{O}$	$N_{R}$	Average PMP				Percentage PMP > 0.75			
		ρ=0	ρ=0.1	ρ=0.3	ρ=0.5	ρ=0	ρ=0.1	ρ=0.3	ρ=0.5
97	215	0.695	0.629	0.905	0.975	51.2	40.8	87.9	97.0
	556	0.836	0.831	0.995	1.000	77.3	76.7	99.4	100
	2,231	0.991	0.991	1.000	1.000	98.9	99.0	100	100
	10,000	1.000	1.000	1.000	1.000	100	100	100	100
218	215	0.709	0.676	0.959	0.993	54.1	49.9	95.0	99.2
	556	0.843	0.840	0.997	1.000	78.3	77.9	99.7	100
	2,231	0.992	0.991	1.000	1.000	98.9	98.9	100	100
	10,000	1.000	1.000	1.000	1.000	100	100	100	100
2,236	215	0.957	0.956	1.000	1.000	95.3	94.3	100	100
	556	0.975	0.974	1.000	1.000	97.4	96.7	100	100
	2,231	0.999	0.998	1.000	1.000	99.9	99.8	100	100
	10,000	1.000	1.000	1.000	1.000	100	100	100	100

To conclude, we do not recommend using the the frequentist and Bayesian FE model as their estimators were biased and hypothesis tests had a high false positive rate in situations where a method’s performance matters most, that is, when the true effect size is zero. HYEMA generally outperforms the Bayes factor based on only the replication data, Edgington’s method, and hybrid with respect to bias, RMSE, and Type-1 error/power. Note, however, that the frequentist FE model slightly outperformed HYEMA in conditions where statistical power of that model’s test approaches 1. Similar to HYEMA, the Bayesian snapshot method performed well in conditions where statistical power was high. Overall, statistical inference using all methods was challenging if the true effect size was small when at least the sum of both study samples sizes was not large.

In the next section, we reanalyzed the SCORE data to examine the replicability of the included original studies. The numerical study indicated several problems with methods to assess replication success for common sample sizes of the original study and replication in the SCORE data. Hence, we did not apply these methods to reanalyze the SCORE data. Instead, we focused on applying the methods for estimating the effect size and testing the null hypothesis of no effect.

### Applying methods to the SCORE data

The reanalysis of the SCORE data consisted of four steps: (1) extracting the correlation estimates and sample sizes of the original studies and replications from the SCORE data, (2) transforming correlation estimates and computing their sampling variances, (3) applying the meta-analytic methods to estimate the effect size and testing the null hypothesis of no effect to the data of the previous step, and (4) summarizing the results of applying the methods.

SCORE included in total 274 effects that were replicated. These studies used a wide variety of designs and consequently different effect size measures were reported. SCORE transformed the effect sizes to correlation coefficients primarily by using the R package “effectsize” [51] whenever this was possible (see p. 152 of Tyner et al. [32] and their supplement at https://osf.io/uqegb/). Correlation coefficients could be computed for 225 out of 274 (82.1%) pairs of the original study and replication. In the first step of the reanalysis, we extracted the correlation coefficients and sample sizes of the 225 pairs of an original study and replication from the publicly available data (https://osf.io/g5sny/files/5xfdy). In the second step, we first multiplied the correlations of the original study and replication by −1 if the correlation of the original study was negative. This was done to ensure that all correlations of the original study were in the same direction. Although SCORE analyzed the correlation coefficients directly, we decided to apply Fisher’s *z* transformation and computed their sampling variance [43]. The reason for applying this transformation is that all methods for estimating the meta-analytic effect sizes and testing the null hypothesis of no effect (except for the Bayes factor based on only the replication and Edgington’s method) assume that the effect size measure follows a normal distribution. However, the correlations in the SCORE data also included other types of correlations than Pearson correlation coefficients, such as point-biserial correlations, for which the usual distributional assumptions underlying Fisher’s *z* transformation do not generally hold. Consequently, how well 1/(N−3) approximated the true sampling variance depends on the type of correlation. We return to this issue in the discussion section.

The third step was applying the methods listed in Table 2 to the Fisher’s *z* transformed correlations. These methods were applied in the same way as described in the Methods section of the numerical study. The hybrid and snapshot method could not be applied to 14 pairs (6.2%), because the correlation of the original study was not significantly different from zero based on a two-tailed test with α=0.05, which is a requirement for these methods. In the last step, we created density distributions of the effect size estimates of the different methods and computed their mean and standard deviation. These results are presented per research field (business, economics, education, political science, psychology, and sociology) as well as averaged across all research fields.

We report aggregated results in the remainder of this section. The results of the individual pairs of original study and replication are available in the supplementary materials (https://osf.io/ktazu). R code of the analyses are available at https://osf.io/gqnwk.

## Results

Fig 4 shows the density distributions of the original study (orange density) and replication (pink density), with the estimated Fisher’s *z* correlations back-transformed to correlations. The density of the replication had its peak substantially closer to zero (mean = 0.153, median = 0.113) than of the original study (mean = 0.300, median = 0.254), which suggests that there was positive bias in the effect size estimator of the original study. The densities of the frequentist and Bayesian FE model (red and green line, respectively), hybrid (black line), and HYEMA (dark blue line) were similar, and all had a peak at approximately 0.07. For all methods, the average estimate was close to 0.2 (see first row of Table 6) and the median estimate was close to 0.15. This implies that effect size estimates were hardly corrected for potential bias by hybrid and HYEMA. The densities of the meta-analysis methods were close to but slightly larger than the density of the replication. The similarity between the estimates of the different meta-analysis models was surprising, because the FE model was expected to overestimate the effect size due to the selection bias by focusing on statistically significant original studies. This can largely be explained by the meta-analysis methods combining information from the original study and replication but assigning more weight to the replication than to the original study due to its larger sample size.

**Fig 4:**
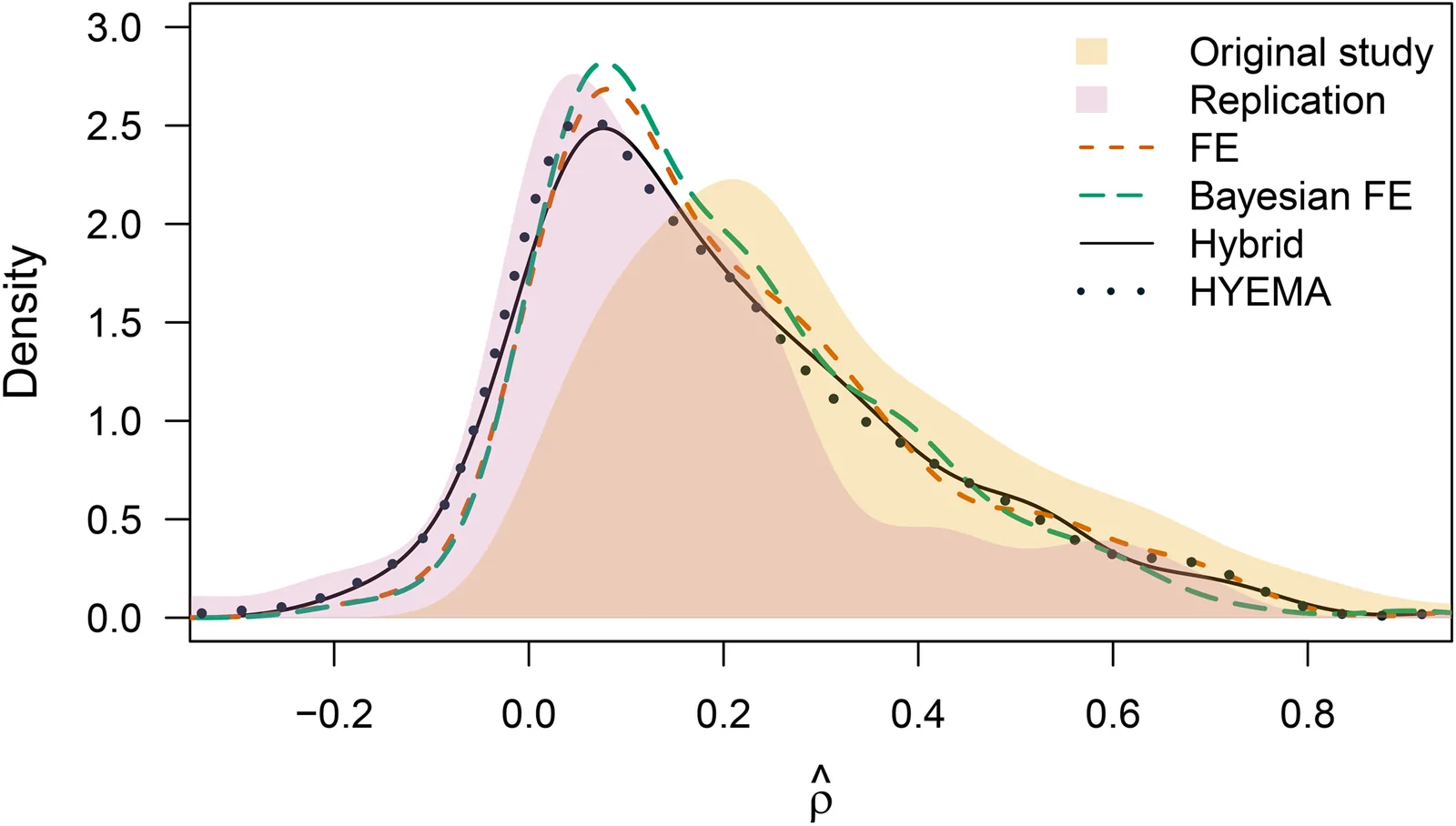
Density of the estimates of the Pearson correlation coefficients of the original study, replication, fixed-effect meta-analysis model (FE), Bayesian fixed meta-analysis model, hybrid method of meta-analysis (Hybrid), and extended hybrid method of meta-analysis (HYEMA). Hybrid could be applied to 211 out of 225 pairs (93.8%).

**Table 6:** Results of applying methods to estimate the effect size when synthesizing the pairs of original study and replication in SCORE.

	# pairs	Mean (SD)		Median (IQR)		Mean (SD)			
		${\hat{\rho }}_{O}$	${\hat{\rho }}_{R}$	$N_{O}$	$N_{R}$	FE	Bayes	Hybrid^a^	HYEMA
Total	225	0.300 (0.205)	0.153 (0.203)	218 (2,139)	556 (2,016)	0.211 (0.192)	0.198 (0.175)	0.200 (0.196)	0.193 (0.201)
Business	36	0.258 (0.117)	0.124 (0.139)	155.5 (112)	519 (598.2)	0.145 (0.116)	0.140 (0.110)	0.136 (0.128)	0.125 (0.121)
Economics	38	0.426 (0.204)	0.243 (0.257)	193.5 (817)	227 (656.2)	0.336 (0.224)	0.311 (0.205)	0.284 (0.251)	0.312 (0.247)
Education	15	0.240 (0.276)	0.135 (0.092)	9,189 (11,248.5)	11,286 (12,121)	0.188 (0.181)	0.183 (0.177)	0.199 (0.170)	0.187 (0.176)
Political science	36	0.286 (0.240)	0.184 (0.202)	5,750 (46,238.5)	6,294 (28,172)	0.241 (0.208)	0.224 (0.183)	0.242 (0.207)	0.234 (0.207)
Psychology	78	0.304 (0.192)	0.134 (0.213)	107 (194.8)	540 (604.4)	0.185 (0.187)	0.173 (0.171)	0.182 (0.193)	0.159 (0.199)
Sociology	22	0.197 (0.154)	0.071 (0.160)	813 (3,014.2)	713.5 (2,154.8)	0.161 (0.126)	0.155 (0.120)	0.144 (0.114)	0.158 (0.135)

*Note:* ${\hat{\rho }}_{O}$ = estimated Pearson correlation coefficient original study, ${\hat{\rho }}_{R}$ = estimated Pearson correlation coefficient replication, $N_{O}$ = sample size of the original study, $N_{R}$ = sample size of the replication, IQR = interquartile range, FE = fixed-effect model, Bayes = Bayesian fixed-effect model

^a^The hybrid method could be applied to 211 out of 225 pairs (93.8%) for which the original study was statistically significant.

The first row in Table 7 presents how often the null hypothesis of no effect was rejected. As expected, more statistically significant results were observed in the original study (93.8%) compared to the replication (58.7%). In the numerical study, the test of the frequentist and Bayesian FE model was too liberal and Edgington’s method was too conservative. This resembled the reanalysis results with the frequentist and Bayesian FE model and Edgington’s method having the largest (81.3% and 76.0%) and smallest (55.6%) percentages of statistically significant results of all methods. The Bayes factor using only the replication data and hybrid and HYEMA had Type-1 error rates close to the nominal rate in the numerical study. The Bayes factor test, hybrid, and HYEMA yielded a statistically significant result in the SCORE data in 64.9%, 59.2%, and 72.9% of the study pairs, respectively. The higher percentage of HYEMA when compared to the Bayes factor test and hybrid may reflect its higher statistical power. HYEMA’s slightly lower percentage (72.9%) than the positively biased frequentist FE model (81.3%) confirmed that bias was relatively small.

**Table 7:** Results of applying methods to test the null hypothesis of no effect when synthesizing the pairs of original study and replication in SCORE.

	# pairs	Percentage statistically significant							
		Orig.	Repl.	FE	Bayes	Edgington	BF repl.	Hybrid^a^	HYEMA
Total	225	93.8	58.7	81.3	76.0	55.6	64.9	59.2	72.9
Business	36	97.2	52.8	72.2	63.9	47.2	47.2	51.4	61.1
Economics	38	97.4	63.2	84.2	84.2	63.2	73.7	51.4	81.6
Education	15	86.7	93.3	100	100	86.7	93.3	100	93.3
Political science	36	97.2	69.4	86.1	86.1	69.4	75.0	80.0	86.1
Psychology	78	91.0	52.6	74.4	67.9	48.7	62.8	50.7	64.1
Sociology	22	90.9	40.9	95.5	77.3	36.4	50.0	55.0	72.7

*Note:* Orig. = original study, Repl. = replication, FE = fixed-effect model, Bayes = Bayesian fixed-effect model, BF = Bayes factor

^a^The hybrid method could be applied to 211 out of 225 pairs (93.8%) for which the original study was statistically significant.

Table 6 also shows the results separately for six research fields and density distributions for each research field are available in the supplemental materials at https://osf.io/ktazu. Comparable estimates were obtained by the methods when applied to pairs within a particular research field. Effect size estimates were on average the smallest for study pairs in business research (approximately 0.14) and the largest in economics (approximately 0.3). The correction for bias by hybrid and HYEMA was the largest for economics. The difference between the frequentist FE model and both hybrid and HYEMA was the largest in this research field as well (estimates 0.336, 0.284, and 0.312 for FE, hybrid, and HYEMA). The largest correction for this research field was probably caused by the larger percentage (31.6%) of original studies with a sample size smaller than 50 in economics compared to all original studies (17.3%). These original studies were more susceptible to selection because overestimation tends to be larger in studies with smaller rather than larger sample sizes. The percentage of statistically significant results were the lowest for business and psychology (approximately 50%) and the highest for education (100%). This aligned with the original study sample sizes that were the smallest for business and psychology and the largest for education.

Table 8 presents the results of applying the snapshot hybrid method to the SCORE data. These results provide evidence regarding the true effect size most likely underlying the pairs of original study and replication. The average PMP are reported for no (ρ=0), a small (ρ=0.1), a medium (ρ=0.3), and a large (ρ=0.5) effect. The results of the average PMP based on all pairs (first row) indicated that no, a small, and a medium effect were all about equally likely. However, there were differences between research fields. No PMP was substantially larger than the others for the study pairs in economics, political science, psychology, and sociology. Evidence for a zero or small effect sizes, based on average PMP, was abundant for business, education, political science, psychology, and sociology, with most evidence for small effect sizes in business research and education. Moreover, evidence for a large effect, either using the average PMP or percentage of PMP larger than 0.75, was small for business research, psychology, and sociology.

**Table 8:** Average posterior model probabilities (PMP) and percentage of PMP larger than 0.75 obtained with the snapshot hybrid method when synthesizing the pairs of original study and replication in SCORE.

	# pairs	Average PMP				Percentage PMP > 0.75				
		ρ=0	ρ=0.1	ρ=0.3	ρ=0.5	ρ=0	ρ=0.1	ρ=0.3	ρ=0.5	Inconclusive
Total	211	0.275	0.321	0.258	0.146	21.3	22.3	20.9	12.8	22.7
Business	35	0.305	0.487	0.193	0.016	25.7	40.0	14.3	0	20.0
Economics	37	0.233	0.134	0.252	0.381	16.2	5.4	21.6	35.1	21.6
Education	13	0.162	0.486	0.195	0.157	15.4	46.2	15.4	15.4	7.7
Political science	35	0.224	0.323	0.236	0.217	20.0	28.6	20.0	17.1	14.3
Psychology	71	0.305	0.309	0.296	0.091	19.7	12.7	22.5	8.5	36.6
Sociology	20	0.356	0.311	0.332	0.000	35.0	30.0	30.0	0	5.0

Results are based on 211 out of 225 pairs (93.8%) for which the original study was statistically significant.

The last column of Table 8 shows that for 22.7% of study pairs the evidence for one effect size was inconclusive (i.e., the PMP was smaller than 0.75 for any of the four effect sizes). The percentage of inconclusive study pairs was low for education (7.7%) and sociology (5.0%), whereas it was relatively high for psychology (36.6%) This likely reflected the larger sample sizes of the study pairs in education and sociology compared to psychology (see second and third column of Table 6).

## Discussion

Replications are important to build a cumulative body of evidence [e.g.[52],. However, there is no considered best way of evaluating the replicability of research. We studied the statistical properties of the majority of replication success methods included in the SCORE project [32]. We also adopted another perspective by meta-analyzing the original study and its replication and interpreting the resulting effect size estimation and statistical inference. This informs the researcher about the magnitude of an effect, and also whether it is significantly different from zero, which are arguably more relevant for theoretical advancement than whether a replication attempt was successful or not. By focusing on effect size estimation in the SCORE data, we also analyzed these data from a new angle that had not yet been considered.

We conducted a numerical study tailored to the characteristics of the SCORE data to evaluate the statistical properties of the different methods. In general, the methods to assess replication success did not perform well, particularly in conditions when performance matters most, that is, when the true effect size is zero or close to zero. Hence, we do not recommend applying these methods. The only exception was the equivalence test that was able to correctly determine replication success if both study sample sizes were large (> 2,000). There was often not enough information in the data (i.e., sample sizes were too small) to draw a reliable conclusion about replication success. This is surprising and telling, because the sample sizes of the replications in the SCORE project were large; the median statistical power to detect the original effect size in the replications was 99.6%. However, for methods assessing replication success to perform well, the sample size of the original study needs to be large as well, which does not frequently occur in practice. Hence, we recommend researchers to be reluctant with applying replication success methods if the true effect size is expected to be zero or small in combination with sample sizes that are not large.

In our numerical study, we also studied the statistical properties of methods for meta-analyzing the original study and replication. The effect size estimators of the traditional frequentist and Bayesian FE model were biased and corresponding hypothesis tests had inflated Type-1 error rates. Dedicated methods to correct for selection bias in the original study, hybrid, HYEMA, and snapshot hybrid, showed low bias and the ability to correctly quantify the magnitude of the effect size. The results also showed that HYEMA was an improvement over hybrid, because it had larger statistical power when testing the null hypothesis of no effect in all conditions. However, it also turned out that the uncertainty in the results of the methods to correct for selection bias was larger than of methods that did not correct for bias, so correcting for bias comes at the cost of less precise estimation. Based on all results, we recommend applying HYEMA to estimate the effect size. FE meta-analysis performed slightly better than HYEMA if statistical power for the original study was high, but this depends on the true effect size that is unknown in practice.

In the reanalysis of the SCORE data, we focused on methods to estimate the effect size and test the null hypothesis of no effect given the unfavorable statistical properties of the replication success methods. When estimating the effect size in the SCORE data, the estimates based on the combination of an original study and replication were closer to zero than those of the original study and in between estimates of the original study and replication. There were only small differences between methods that corrected and did not correct for bias. The estimates averaged over all pairs of original study and replication were close to 0.2 and the modes of the estimates were approximately 0.07, signaling many true effect sizes that were of zero or small magnitude. The lack of correction for bias was likely caused by the generally substantially larger sample size of the replication versus the original study, resulting in more weight of the unbiased replication in a meta-analysis. However, we do not recommend to only apply methods that do not correct for bias, because our numerical study showed that the Type-1 error rate of these methods could be severely inflated. Hence, not surprisingly, these methods had a higher percentage of statistically significant findings than the methods that aim to correct for bias in the reanalysis of the SCORE data.

We also presented the results of the reanalysis of the SCORE data separately for each research field. Most important to mention is that results within each field were diverse; with the exception of business and sociology where no large effects were found. The snapshot hybrid method found evidence for zero, small, medium, and large effects in study-pairs in the fields of economics, education, political science, and psychology. Hence, one should always be careful with general statements on the magnitude of the studies’ true effect size in a field. Evidence for zero or small effect sizes (i.e., a correlation of 0.1 or smaller) was predominant (> 50%) in all research fields except for economics, and with strongest evidence for zero and small effects in business. Because the distinction between a zero and small true effect size is most important for both theoretical and practical reasons and these true effects are most challenging for statistical methods to perform well, designing (original and replication) studies with sufficiently large sample sizes is of utmost importance when a zero or small true effect size is expected. Larger sample sizes then also result in less biased effect size estimators of traditional statistical methods, which we observed for original studies in economics that often use small sample sizes compared to other fields. As using large sample sizes for medium or large true effect sizes may entail a waste of resources, we support [53] and others to recommend conducting power analyses for study sample size justification.

An important conclusion based on our findings is that there may not be sufficient information in a single original study and a single replication to draw definite conclusions. This observation is in line with previous work [e.g., 12,21–23,25]. At least part of the solution to this is conducting multiple replication studies. This can be set up as a registered replication report [54], in which an original study is replicated in multiple labs using the same experimental procedures. A registered replication report has the additional advantage that it allows for studying whether characteristics of the labs influence the replicability. Another solution is not to consider the original study, particularly when it has a small sample size, as it yields a biased effect size estimator due to being selected for statistical significance either by the researchers who replicate a study and/or publication bias [13]. This solution is backed up by our analysis of the SCORE data that generally showed similar estimates of the replication and meta-analysis methods correcting for statistical significance in the original study. However, note that that the precision is usually higher of these meta-analysis methods because of using more information than only the replication.

We followed the implementation of the methods used in SCORE for both the numerical study and the reanalysis of the SCORE data. The only exception was that we used different Bayes factor thresholds. In SCORE [32], different Bayes factor thresholds were used for different methods. This made comparisons between methods challenging, so we implemented a Bayes factor of three for all methods such that three times more evidence for one hypothesis was considered replication success. A Bayes factor threshold of three is a commonly used threshold reflecting moderate evidence in favor of a hypothesis [45].

The numerical study was tailored to characteristics of the SCORE data. An advantage of this is that the results of the numerical study are directly applicable to the SCORE data. A disadvantage of this approach is that the generalizability of the findings of the numerical study might be limited. Future research might want to conduct an analysis with a more general set of conditions, although we believe that the methods assessing consistency remain flawed in case of selection of a statistically significant original study.

Another limitation is that our calculation of the percentages of replication success and of the average effect size did not consider that some studies (32.0%) yielded multiple effects. These results may be biased if original studies yielding multiple effects systematically obtain higher (or lower) replication success or effect size than unique original studies. Tyner et al. [32] reported results both at the level of individual effects and at the level of the paper, and found only small differences between the two; median Pearson correlations of 0.25 (paper level) and 0.24 (effect level) for original studies, and 0.10 (paper level) and 0.13 (effect level) for replications. Hence, the violation of the independence assumption in our analyses is expected to affect our results only to a small extent and is unlikely to alter our conclusions.

A final limitation of this study is that we reanalyzed the shared data by SCORE. By reanalyzing the shared data by SCORE, we also adopted their effect size transformations to correlation coefficients. We subsequently applied Fisher’s *z* transformation to the data in order to work with an approximately normally distributed effect size measure in our analyses. These effect size transformations often hinge on strong assumptions and yielding effect size estimates with unknown distributional properties [55]. Applying the transformations may have introduced bias in the results but also helped summarizing the results as most effect sizes were on a common scale.

To conclude, we have shown that not all replication success methods had good statistical properties for the SCORE data. This warrants for not routinely applying replication success methods, but only applying methods that are expected to have good properties for the analyzed data. The decision on what methods to apply can be based on our numerical study or on available literature that has examined the properties of the methods [e.g., 22,27,28]. We hope for a continued attention for replications and the development of replication success methods that overcome the deficiencies of the existing methods.
